# Assessment of treatment patterns and patient awareness in atrial fibrillation patients using non-vitamin K antagonist oral anticoagulants (ASPECT-NOAC)

**DOI:** 10.1016/j.ijcha.2022.100989

**Published:** 2022-03-02

**Authors:** Özer Badak, Ali Rıza Demir, Tugay Önal, Taylan Akgün, Osman Can Yontar, Ömer Şatıroğlu, Hakan Duman, Ertuğrul Okuyan, Mehmet Melek, İbrahim Etem Dural

**Affiliations:** a Dokuz Eylül University, Faculty of Medicine, Department of Cardiology, İzmir, Turkey; b University of Health Sciences, Istanbul Mehmet Akif Ersoy Thoracic and Cardiovascular Surgery Training and Research Hospital, Department of Cardiology, Istanbul, Turkey; c Pfizer, Medical Department of Internal Medicine, Istanbul, Turkey; d Kartal Koşuyolu High Specialty Educational and Research Hospital, Department of Cardiology, Istanbul, Turkey; e Samsun Training and Research Hospital, Department of Cardiology, Samsun, Turkey; f Recep Tayyip Erdoğan University, Faculty of Medicine, Department of Cardiology, Rize, Turkey; g Health Sciences University, Bağcılar Training and Research Hospital, Department of Cardiology, Istanbul, Turkey; h Bursa High Specialty Education and Research Hospital, Department of Cardiology, Bursa, Turkey; i Afyonkarahisar Health Sciences University, Faculty of Medicine, Department of Cardiology, Afyonkarahisar, Turkey

**Keywords:** Atrial fibrillation, Non-vitamin K oral anticoagulants, NOAC, Treatment patterns, Patient characteristics, Patient knowledge, Treatment continuity

## Abstract

**Background and Aim:**

Despite the advances in oral anticoagulation with NOACs, careful patient and dose selection is required with NOAC therapy. Our study aimed to assess treatment patterns of NOACs in AF along with patients’ continuity to NOAC treatments in first year, and their knowledge level of AF and NOAC treatment.

**Methods:**

ASPECT-NOAC was designed as an observational, prospective, and multicenter study. AF patients who were prescribed NOACs within last four months were recruited from 34 outpatient cardiology clinics covering all geographic regions of Turkey. Baseline data were collected initially whereas patient awareness was evaluated at 3 to 4 weeks. Final study visit was performed at 12 months.

**Results:**

In total, 991 patients were included to the study. Mean ± standard deviation of age was 69.4 ± 10.2 years and 53.0% of patients were female. Mean duration from AF diagnosis was 24.9 ± 50.9 months. Mean CHA_2_DS_2_-VASc and HAS-BLED scores were 3.1 ± 1.5 and 1.6 ± 1.1, respectively. AF disease and NOAC treatment knowledge levels were found to be 48.9 ± 23.1% and 73.0 ± 19.3%, respectively. Among reduced dose users 71.4% of patients were prescribed inappropriate reduced doses. Through the study follow-up, 32 patients (3.2%) deceased and NOAC therapy was discontinued in 74 patients (8.7%).

**Conclusion:**

AF patients who recently started NOAC treatment in Turkey were found to have variable knowledge about their disease and anticoagulation treatment. It was observed that most of the patients continued the NOAC treatment throughout the study. Reduced dosing of NOACs was common, which was associated with higher baseline risk for bleeding as well as stroke.

## 1 Introduction

Atrial fibrillation (AF) is the most common sustained cardiac rhythm disturbance, and its prevalence increases with age [1] (Lip2016). Hemodynamic impairment and cardioembolism related to AF result in significant morbidity and mortality [2] (Hindricks2021). Prevention of ischemic stroke is the main objective of AF management [3] (Wolf1991). For many years, vitamin K antagonists (VKAs) have been the only oral anticoagulant drugs available for clinical use in the prevention of venous and arterial thromboembolic events [4] (Ageno2012). Several factors, such as food and drugs, that affect the pharmacokinetics of VKAs, can cause deviations from their narrow therapeutic window, by increasing the bleeding or thrombosis risk and complicating long-term use of VKAs [5] (Vranckx 2018).

Non-vitamin K antagonist oral anticoagulants (NOACs) were introduced within the last decade for the prevention of AF-related stroke. Drugs in two different classes, direct inhibitors of thrombin and factor Xa inhibitors, have since been approved worldwide. NOACs provide similar or better stroke prevention in AF with an improved safety and easier management profiles [6] (Chan2016). Despite the advances in oral anticoagulation with NOACs for the prevention of stroke in AF patients, careful patient and dose selection is required with NOAC therapy. Use of NOACs with lower or higher than recommended doses have been reported as an important problem, resulting in suboptimal management of stroke prevention [7], [8], [9] (Garcia2019, Barra2016, Pattullo2016).

Previous observational studies conducted in Turkey highlighted high rates of inappropriate dosing in NOAC use. Cardiologists' adherence to international guidelines were found to be suboptimal and about 40% of AF patients were administered with inappropriately lower or higher NOAC doses. Adherence of AF patients to NOAC therapy was also poor. Low medication adherence was recorded in half of AF patients on NOAC treatment [10], [11] (Basaran2016, Emren2018). Taken together, these results urge continuous efforts to optimize management of AF with NOAC medications in Turkey. Our current study aimed to assess treatment patterns of NOACs for the prevention of stroke in AF along with patients’ continuity to NOAC treatments in first year, and their knowledge level of AF as a disorder and NOAC treatment.

## 2 Methods

### 2.1 Study design and patients

ASPECT-NOAC was designed as an observational, prospective, longitudinal, and multicenter study. AF patients were recruited from 34 outpatient cardiology clinics of state, university, private, and research hospitals covering all geographic regions of Turkey. Adult (≥18 years) AF patients with an ongoing NOAC treatment (apixaban, dabigatran, edoxaban or rivaroxaban), who had been initiated NOAC treatment within the last four months, were included. Exclusion criteria were cognitive impairments or difficulty in understanding as assessed by an investigator and participation in another study within the last six months. Patient enrollment was conducted between January 2018 and December 2018. Study design was approved by the ethics committee of the coordinating study site (Decision No.: 2917/20–04, Date: 30.11.2017). All patients were informed about the study and provided a written consent for study participation before any study related activities.

### 2.2 Objectives and data collection

Primary objective was the evaluation of treatment patterns and characteristics of AF patients under NOAC treatment for the prevention of stroke. Secondary objectives were the assessment of patient knowledge of AF and NOAC treatment, continuation of NOAC treatment at 12 months, and factors related with NOAC medication continuity. Baseline demographics, clinical and medication history, and presence of risk factors were collected via an electronic case report form at initial enrolment (baseline visit) whereas patient awareness (i.e. knowledge levels) was evaluated via a telephone interview at 3 to 4 weeks. Final study visit was performed at 12 months and NOAC treatment modifications and mortality were recorded. Baseline CHA_2_DS_2_-VASc (congestive heart failure or left ventricular dysfunction, hypertension, age 65–75 years, diabetes mellitus, vascular disease, female sex [1 point for presence of each], thromboembolism or stroke history, age ≥ 75 years [2 points for presence of each]) and HAS-BLED (hypertension, abnormal renal function, abnormal liver function, stroke, bleeding history or predisposition, labile international normalized ratio, elderly [age > 65 years], drugs predisposing to bleed, alcohol use [1 point for presence of each]) scores were calculated to assess stroke and major bleeding risk, respectively. Appropriateness of daily NOAC doses were determined via the dose modification criteria stated in approved summaries of NOAC product characteristics (SmPC) for each patient. Both 150 mg and 110 mg BID of dabigatran were considered appropriate doses, since dabigatran does not have specific dose reduction criteria for both doses in its clinical trials. Recommended dose-reduction criteria of dabigatran were age >_80 years, concomitant use of verapamil, or increased bleeding risk according to AF guidelines [2] (Hindricks2021).

Patient awareness for AF and NOAC treatment was measured with modified Jessa Atrial Fibrillation Knowledge Questionnaire (JAKQ) and percentage of correct answers were calculated separately for AF and NOAC treatment [12] (Desteghe2016) (Table 1).

**Table 1:** Atrial fibrillation and non-vitamin K antagonist oral anticoagulants knowledge questionnaire.

**Questions for atrial fibrillation awareness**	**Correct answer**
Is the blood thinner medication you are taking given to protect you from stroke?	Yes
Does the rhythm disturbance in your heart cause blood clots to form, which can lead to stroke?	Yes
Is atrial fibrillation a condition where the heart beats irregularly and often faster than normal?	Yes
Is atrial fibrillation always accompanied by symptoms?	No
Can patients detect atrial fibrillation by taking their pulse regularly?	Yes
Can being overweight exacerbate atrial fibrillation?	Yes
Can your medication prevent atrial fibrillation permanently?	No
**Questions for non-vitamin K antagonist oral anticoagulants awareness**	
Do you have to take your blood thinner medication even you do not feel palpitation?	Yes
Is it important to take your blood thinner medication at the same time every day?	Yes
Can you take painkillers while on blood thinner medication?	No
When you have forgotten to take your blood thinner medication dose, should you take the missed dose even if the next dose is due in short time?	No
Can the blood thinner medication you take cause various spontaneous bleeding in your body or can it be difficult to stop bleeding when you are injured?	Yes
If you need an operation, should you consult your physician who has prescribed the blood thinner medication?	Yes
Should you use blood thinner medication for a lifetime?	Yes

### 2.3 Sample size and statistical analyses

Assuming an average response rate of 50% with a 5% margin of error and 99% confidence interval (CI), the required sample size was calculated to be 662 as a representative number of AF patients who have been using NOACs in Turkey. Addition of an extra 50% to allow for probable dropouts resulted in a final sample size of 993. The sample size was calculated by PS software (PS Power and sample size calculation V3.1.2). Patient knowledge levels were evaluated for subgroups of gender, education level, and income level while correlation analyses were performed for age and BMI. Normality test of numerical variables was performed by Shapiro-Wilk test and descriptive statistics were presented as mean ± standard deviation (SD). Categorical variables were expressed as numbers and percentages. Chi-square test was used for the comparison of nominal data. In the comparison of two independent groups, independent samples *t*-test or Mann-Whitney *U* test were used as appropriate. For the comparison of multiple groups, Kruskal-Wallis or ANOVA tests were used where appropriate. Mann-Whitney *U* test and Bonferroni correction were used for post-hoc analysis of Kruskal-Wallis test. Tukey test was used for post-hoc analysis of ANOVA. Correlation analyses were performed with Spearman rank correlation. Binary logistic regression analysis was carried out to evaluate the effects of baseline characteristics on NOAC therapy modifications (discontinuation, switch or daily dose adjustment) during study follow-up and odds ratio (OR) and 95% CI were estimated. Effect size was calculated as Cohen’s d. SPSS (version 23) and Jamovi (version 1.0.8) were used in statistical analysis. Significance level (p value) was considered as 0.05 and Bonferroni adjustment was applied in post-hoc tests.

## 3 Results

**Fig. 1:**
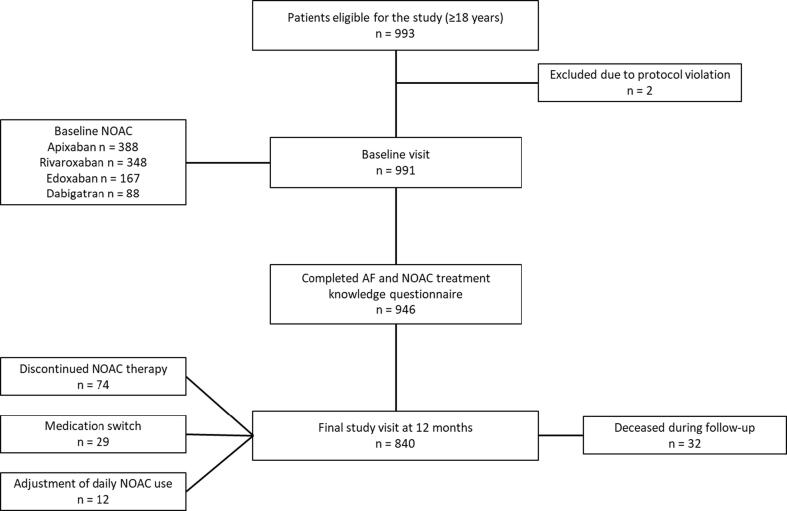
Patient flowchart.

In total, 993 patients were enrolled to the study. Two patients were excluded from the analyses due to protocol violation and data from the baseline visits of 991 patients were included. Of these, 840 patients (84.8%) attended the final study visit at 12 months. Patient flowchart is shown in Fig. 1.

Mean age was 69.4 ± 10.2 years and 525 patients (53.0%) were female. Female patients were significantly older (mean ± SD = 70.1 ± 9.8, p = 0.021) and mean ± SD age of male patients was 68.6 ± 10.5. Female patients had significantly higher BMI levels (p < 0.001).

Mean duration from AF diagnosis was 24.9 ± 50.9 months. Previous stroke history was positive in 126 patients (12.7%). Mean CHA_2_DS_2_-VASc and HAS-BLED scores were 3.1 ± 1.5 and 1.6 ± 1.1, respectively. Demographic and clinical characteristics of patients are shown in Table 2.

**Table 2:** Baseline patient characteristics.

**Characteristic**	**All patients (n = 991)**
**Age, years, mean (SD)**	69.4 (10.2)
**Gender, female, n (%)**	525 (53.0)
**Body mass index, kg/m^2^, mean (SD)**	29.4 (5.7)
**Duration from AF diagnosis, month, mean (SD)**	24.9 (50.9)
**Atrial fibrillation type, n (%)**	
Permanent (chronic)	482 (48.6)
Paroxysmal	280 (28.3)
Newly diagnosed	136 (13.7)
Persistent	58 (5.9)
Long-term persistent	35 (3.5)
**Education level, n (%)**	
Illiterate	247 (24.9)
Primary school	535 (54.0)
Secondary school	88 (8.9)
High school	82 (8.3)
University	39 (3.9)
**Occupation, n (%)**	
Unemployed	501 (50.6)
Employed	48 (4.8)
Retired	442 (44.6)
**Income perception, n (%)**	
Poor	20 (2.0)
Below average	262 (26.4)
Average	677 (68.3)
Above average	32 (3.2)
**Cardiovascular risk factor, n (%)**	
Smoking	129 (13.0)
Alcohol abuse	26 (2.6)
Sedentary lifestyle	352 (35.5)
**CHA_2_DS_2_-VASc score, mean (SD)**	3.1 (1.5)
**HAS-BLED score, mean (SD)**	1.6 (1.1)
**Comorbidity (>10%), n (%)**	
Hypertension	681 (68.7)
Coronary heart disease	357 (36.0)
Diabetes mellitus	313 (31.6)
Valvular heart disease	184 (18.6)
Dyslipidemia	175 (17.7)
Chronic obstructive pulmonary disease	142 (14.3)
Cardiomyopathy	104 (10.5)

AF, atrial fibrillation; NOAC, non-vitamin K antagonist oral anticoagulant; SD, standard deviation

AF and NOAC treatment knowledge questionnaire were completed by 946 patients (95.5%). The modified JAKQ AF and NOAC treatment knowledge level scores were found to be 48.9 ± 23.1% and 73.0 ± 19.3%, respectively. AF knowledge level was varied and associated with education level (Cohen's d = 0.186, p = 0.017). However, in post-hoc analysis, there was no difference between educational level groups. AF knowledge level was associated with patient age (r = −0.104, p = 0.001) and BMI (r = −0.179, p < 0.001). NOAC knowledge level was higher in males (Cohen's d = 0.187, p = 0.003) and associated with age (r = −0.081, p = 0.013) and BMI (r = −0.179, p < 0.001). While the p value shows a statistically significant difference between these parameters, the r value shows a weak negative correlation. There was no significant difference of both AF and NOAC treatment knowledge level score for perception of income level (p > 0.05) and discontinuation of medication (p > 0.05).

At baseline, apixaban was the most commonly used (n = 388, 39.2%) NOAC, followed by rivaroxaban (n = 348, 35.1%), edoxaban (n = 167, 16.9%), and dabigatran (n = 88, 8.9%). CHA_2_DS_2_-VASc and HAS-BLED scores were similar among NOAC medication subgroups (p > 0.1 for both scores). Only 4.2% (n = 42) patients were treated with standard and/or higher doses of NOACs despite reduced doses were recommended by SmPC. Subsequent comparisons were performed for undertreated patients who were using inappropriately reduced doses. Excluding dabigatran users, 21.7% (n = 196) of patients were prescribed reduced doses. Among reduced dose users, 71.4% (n = 140) of patients were prescribed inappropriate reduced doses (Table 3).

**Table 3:** NOAC treatments and dose levels.

NOAC Treatments	Reduced dose	Standard dose*	Higher dose	Total (%)
Apixaban	82	306	0	388 (39.2%)
Rivaroxaban	84	261	3	348 (35.1%)
Edoxaban	30	135	2	167 (16.9%)
Dabigatran	0	88	0	88 (8.9%)
Total	196 (19.8%)	790 (79.7%)	5 (0.5%)	991 (100%)

*For apixaban, 10 mg/day; for rivaroxaban, 20 mg/day; for edoxaban, 60 mg/day and for dabigatran, 220–300 mg/day is accepted as standard doses.

These undertreated patients were found to be significantly older (median 76.7 vs 68.9 years, p < 0.001), have lower hemoglobin levels (12.7 ± 1.8 vs 13.1 ± 1.8 g/dl, p = 0.02) and have higher serum creatinine levels (1.0 ± 0.3 vs 0.94 ± 0.3 mg/dl, p = 0.02). CHA_2_DS_2_-VASc and HAS-BLED and scores were significantly higher in these patients (3.5 ± 1.4 vs 3.0 ± 1.5 and 1.9 ± 1.1 vs 1.5 ± 1.1, respectively; p < 0.001 for both). Majority (95.2%) of the undertreated patients at baseline were still administered with lower than recommended doses of NOACs at 12 months. Through the study follow-up, 32 patients (3.2%) deceased. While baseline CHA_2_DS_2_-VASc and HAS-BLED scores and AF and NOAC knowledge levels of the deceased were similar to those alive at 12 months (p = 0.089, 0.079, 0.784, and 0.242, respectively), deceased patients were found to be older (mean 75.2 ± 11.5 vs 69.1 ± 10.0 years, p = 0.001) and inappropriately undertreated more frequently (18.8% vs 3.6%, p < 0.001).

**Fig. 2:**
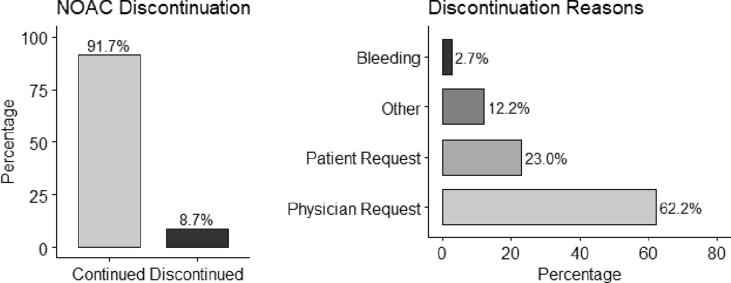
NOAC treatment discontinuations and their reasons.

Over the study follow-up, NOAC therapy was discontinued in 74 patients (8.7%). NOAC medications according to medication discontinuation subgroups and recorded reasons for medication discontinuation during the study are shown in Fig. 2.

Duration from AF diagnosis was shorter in patients who discontinued NOAC therapy (21.7 ± 41.7 vs 26.2 ± 53.7 months, p = 0.017). Medication switch between NOACs was recorded in 29 patients (2.9%). Adjustment of daily NOAC dose was performed for 12 patients (1.2%). In regression analysis, only the presence of a cardiovascular risk factor was found to be an independent indicator for any modification in NOAC therapy over 12 months (OR:1.69, 95% CI: 1.07–2.67; p = 0.026).

## 4 Discussion

ASPECT-NOAC study evaluated patient characteristics, disease and novel anticoagulation therapies knowledge levels, and treatment continuation in AF patients who recently initiated NOAC treatment. Patient characteristics were found to be similar to our previous national study on NOAC use (Table 1): AF patients were elderly at high risk of stroke with slight female dominance, low educational status, and significant cardiovascular risk factors and comorbidities (10) (Basaran2016).

Average AF knowledge level was found to be less than 50% with a wide margin of variability among patients. Lower education level, advanced patient age, and higher BMI were found to be associated with lower AF awareness, albeit only weakly. Lower AF knowledge levels among older patients and patients with lower educational status have been previously reported [13], [14] Sedney2021, McCabe2008). Another reason why elderly patients with AF have lower knowledge could be that they have more than one comorbidity with age. The most common comorbidities seen in patients with AF were hypertension, coronary heart disease and diabetes mellitus in this study. Most common cardiovascular risk factor was sedentary lifestyle (Table 2). Contrary to the study of Sedney et al., the number of female patients in this study was slightly higher than male patients and also the mean age of female patients was slightly higher (female mean ± SD = 70.1 ± 9.8, male mean ± SD = 68.6 ± 10.5, p = 0.021). NOAC knowledge level was higher in males and associated with lower mean age (Cohen’s d = 0.187, p = 0.003). NOAC medication knowledge levels were found to be higher than AF knowledge levels. Recent initiation of NOACs in our study population could account for this finding. Advanced age and higher BMI, however, were still associated with lower NOAC knowledge levels. Higher levels of anticoagulant medication knowledge in AF patients were reported to be related with better medication adherence [15] (Rolls2017). In our study, AF disease and NOAC treatment knowledge levels is not significantly different between the patients who discontinued their medication and those who continued. NOAC medication adherence was found to be low in about half of AF patients in Turkey and nonadherence was associated with higher stroke and bleeding risks [11] (Emren2018). Educating AF patients is considered as an important aspect of AF management for better medication adherence, and safer and more effective anticoagulant use [16] (Lane2015). Continued education of AF patients should be considered as a routine practice in AF management. Use of surveys or individualized and targeted messaging could be implemented for the AF educational sessions [17], [18] (Toscos2020, Desteghe2019).

Most commonly administered NOAC treatments were apixaban and rivaroxaban (n = 388, 39.2% and n = 348, 35.1% patients, respectively). Of all, 79.7% of patients were taking NOACs in standard dose levels (Table 3). Previous studies in Turkish AF patient population had showed that about 30% of AF patients were undertreated while 7–10% were overtreated with NOACs [10], [19], [20] (Basaran2016, Belen2015, Basaran2017). We found that one fifth of AF patients were prescribed with inappropriate doses of NOAC medications and almost all of these patients were undertreated. This finding indicates that dose selection of NOACs has improved considerably over the last five years and overdosing are seen less frequently. Yet, a substantial number of AF patients continue to receive inadequate NOAC doses in Turkey. Undertreated patients were found to be older and had higher stroke and bleeding risk. Once initiated, prescriptions with inadequate NOAC doses persisted over a year and only rarely changed in our study.

Inappropriate dosing of NOACs has been associated with increased thromboembolism and mortality in AF patients [21], [22], [23] (Godino2020, Camm2020, Steinberg2018). In accordance with these publications, we found that the rate of undertreatment with NOACs was higher in the deceased. Even though the deceased was older, and the study was not powered for a survival analysis, the association between inappropriate low dose anticoagulant use and increased mortality warrants vigilance. Current European guidelines for the management of AF explicitly state that a high bleeding risk should not be a reason to withhold effective oral anticoagulation [2] (Hindricks2021). Issues and recommendations related to reduced dosing for AF patients with NOACs have been addressed as well [2], [19], [24], [25] (Hindricks2021, Belen2015, Dillinger2018, Chan2019). Dose adjustments or switch between NOACs were observed to be rare while NOAC therapy was discontinued in 8.7% (n = 74) and was continued in 91.3% (n = 780) of AF patients within a year of follow-up. Physician request was stated as the reason for discontinuation in majority of these cases (n = 46, 62 %) and the other reasons were patient request (n = 17, 23 %), bleeding (n = 2, 3 %) and other reasons (n = 9, 12 %) (Fig. 2). These are patient-reported outcomes, and we were not able to determine root causes of anticoagulant cessation in these patients.

### 4.1 Limitations

Some limitations apply to our study. Primarily designed as an observational study with descriptive outcomes, patient awareness for AF and NOAC treatments were not followed for knowledge retention over time. We did not record concomitant antiplatelet and nonsteroidal anti-inflammatory medication use of patients, which might be of importance for thoughtful undertreatment with NOAC therapies. Detailed reasons of NOAC discontinuation were not proactively sought either. Finally, we did not apply correction for multiple statistical comparisons within patient subgroups. Thus, our results should be interpreted with caution and regarded as findings for hypothesis generation in future studies. We believe that studies focusing on [1] increasing patient knowledge of AF and NOAC therapies, [2] reasons for reduced dosing and discontinuation of novel oral anticoagulants, and [3] their relations with all-cause mortality are warranted to optimize AF management in Turkey.

## 5 Conclusions

This study aimed to evaluate correctness of NOAC dose regimen in AF patients, thus identifying physician's compliance with the guidelines’ recommendations. AF patients who recently started NOAC treatment in Turkey were found to have variable knowledge about their disease and anticoagulation treatment and there were different NOAC and AF knowledge gaps in these patients. Reduced dosing of NOACs was common, which was associated with higher baseline risk for bleeding as well as stroke. Guidelines and product recommendations should be followed more strictly for optimal NOAC dosing in AF patients.

## Funding

This study was funded by Pfizer.

## Declaration of Competing Interest

The authors declare that they have no known competing financial interests or personal relationships that could have appeared to influence the work reported in this paper.
