# Supplementary material for: Assessment of treatment patterns and patient awareness in atrial fibrillation patients using non-vitamin K antagonist oral anticoagulants (ASPECT-NOAC)
Source: Int J Cardiol Heart Vasc. 2022 Mar 2;39:100989. doi: 10.1016/j.ijcha.2022.100989 (PMC8897699; doi:10.1016/j.ijcha.2022.100989)
Supplement: Supplementary data 1 [file mmc1.docx]

**SUPPLEMENTARY APPENDIX**

**Study Title:** Assessment of Treatment Patterns and Patient Awareness in Atrial Fibrillation Patients Using Non-Vitamin K Antagonist Oral Anticoagulants (ASPECT-NOAC)

**STUDY PERSONNEL**

**NIS (Non-Interventional Study) Lead:** Tugay Onal, MD

**NIS Former Lead:** Serdar Alkan, MD

**NIS Project Managers:** Yağmur Pak, Pharm.; Z. Aslı Dülgeroğlu

**Contract Research Organizations:** Pleksus CRO Inc., ETHIC Medical Research Ltd

**INVESTIGATORS**

**Primary Individual Responsible Investigator:** Prof. Dr. Özer Badak, Dokuz Eylül University School of Medicine, Department of Cardiology

| **NO** | **Study Center** | **Investigator** | **Role** |
| --- | --- | --- | --- |
| **1** | Dokuz Eylül Üniversitesi Tıp Fakültesi Hastanesi | Prof. Dr. Özer Badak | PI (Primary Investgator) |
|  |  | Dr. Deniz Çırgamış | SubI (Sub-Investigator) |
|  |  | Dr. Ahmet Anıl Başkurt | SubI |
| **2** | Türkiye Yüksek İhtisas Eğitim ve Araştırma Hastanesi | Prof. Dr. Dursun Aras | PI |
|  |  | Dr. Ertan Ekici | SubI |
| **3** | Dr. Siyami Ersek Göğüs Kalp ve Damar Cerrahisi Eğitim ve Araştırma Hastanesi | Prof. Dr. Ahmet Taha Alper | PI |
|  |  | Dr. Duygu İnan | SubI |
| **4** | Hacettepe Üniversitesi Tıp Fakültesi Hastanesi | Yrd. Doç. Dr. Uğur Canpolat | PI |
|  |  | Dr. Cem Çöteli | SubI |
| **5** | Cumhuriyet Üniversitesi Tıp Fakültesi Araştırma ve Uygulama Hastanesi | Prof. Dr. Mehmet Birhan Yılmaz | PI |
| **6** | Mersin Üniversitesi Hastanesi | Doç. Dr. Ahmet Çelik | PI |
|  |  | Dr. Mert Koray Özcan | SubI |
| **7** | Sivas Numune Hastanesi | Uz. Dr. Hasan Ata Bolayır | PI |
| **8** | Kahramanmaraş Sütçü İmam Üniversitesi Araştırma ve Uygulama Hastanesi | Prof. Dr. Tuna Katırcıbaşı | PI |
|  |  | Dr. Murat Kerkütlüoğlu | SubI |
| **9** | Bağcılar Eğitim ve Araştırma Hastanesi | Doç. Dr. Ertuğrul Okuyan | PI |
|  |  | Dr. Kamil Gülşen (left study at 03.04.2019) | SubI |
|  |  | Dr. Emre Melik Faideci | SubI |
|  |  | Dr. Hanife Abanus | SubI |
|  |  | Uzm. Dr. Sinan Varol | SubI |
| **10** | Trakya Üniversitesi Sağlık Araştırma ve Uygulama Merkezi | Yrd. Doç. Dr. Servet Altay | PI |
|  |  | Dr. Çağlar Kaya | SubI |
| **11** | Bakırköy Dr. Sadi Konuk Eğitim ve Araştırma Hastanesi | Doç. Dr. İbrahim Faruk Aktürk | PI |
|  |  | Uzm. Dr. Veli Polat | SubI |
| **12** | Ahi Evren Göğüs Kalp ve Damar Cerrahisi Eğitim ve Araştırma Hastanesi | Doç. Dr. Levent Korkmaz | PI |
|  |  | Uzm. Dr. Ömer Faruk Çırakoğlu | SubI |
|  |  | Uzm. Dr. Selim Kul | SubI |
| **13** | Namık Kemal Üniversitesi Sağlık Uygulama ve Araştırma Hastanesi | Yrd.Doç. Dr. Demet Özkaramanlı Gür | PI |
|  |  | Doç. Dr. Şeref Alpsoy | SubI |
| **14** | Marmara Üniversitesi Tıp Fakültesi Pendik Eğitim ve Araştırma Hastanesi | Doç. Dr. Ahmet Altuğ Çinçin | PI |
|  |  | Doç. Dr. Yusuf Emre Gürel | SubI |
|  |  | Uzm. Dr. Ahmet Anıl Şahin | SubI |
| **15** | Samsun Eğitim ve Araştırma Hastanesi | Doç. Dr. Osman Can Yontar | PI |
|  |  | Doç. Dr. Uğur Arslan | SubI |
| **16** | Dışkapı Yıldırım Beyazıt Eğitim ve Araştırma Hastanesi | Doç. Dr. Murat Tulmaç | PI |
|  |  | Uz. Dr. Mehmet Erat | SubI |
|  |  | Dr. Alperen Taş | SubI |
|  |  | Dr. Khudaverdi Khojamguliyev | SubI |
| **17** | İstanbul Medeniyet Üniversitesi Göztepe Eğitim ve Araştırma Hastanesi | Uz. Dr. Gönül Açıksarı | PI |
|  |  | Dr. Fatma Betül Özcan | SubI |
| **18** | Antalya Eğitim ve Araştırma Hastanesi | Doç. Dr. Göksel Çağırcı | PI |
|  |  | Dr. Emre Asiltürk | SubI |
| **19** | Akdeniz Üniversitesi Hastanesi | Doç. Dr. Refik Emre Altekin | PI |
|  |  | Dr. Ali Yaşar Kılınç | SubI |
| **20** | Adana Numune Eğitim ve Araştırma Hastanesi Seyhan Uygulama Kardiyoloji Merkezi | Uzm. Dr. Durmuş Yıldıray Şahin | PI |
|  |  | Gonca Gümüş | SC |
| **21** | Özel Adana Ortadoğu Hastanesi | Doç. Dr. Ahmet Oytun Baykan | PI |
| **22** | İstanbul Üniversitesi Kardiyoloji Enstitüsü | Prof. Dr. Murat Ersanlı | PI |
|  |  | Dr. Fevziye Burcu Topçu | SubI |
| **23** | Şişli Hamidiye Etfal Eğitim ve Araştırma Hastanesi | Doç. Dr. Kadriye Kılıçkesmez | PI |
|  |  | Uzm. Dr. Kudret Keskin | SubI |
| **24** | Atatürk Üniversitesi Tıp Fakültesi Sağlık Araştırma ve Uygulama Merkezi | Doç. Dr. Muhammed Hakan Taş | PI |
|  |  | Dr. Sidar Şiyar Aydın | SubI |
| **25** | Afyon Kocatepe Üniversitesi Hastanesi | Prof. Dr. Ersel Onrat | PI |
| **26** | Bursa Yüksek İhtisas Eğitim ve Araştırma Hastanesi | Doç. Dr. Mehmet Melek | PI |
|  |  | Doç. Dr. Hasan Arı | SubI |
|  |  | Dr. Berat Uğuz | SubI |
|  |  | Doç. Dr. Selma Kenar Tiryakioğlu | SubI |
|  |  | Dr. Selma Arı | SubI |
| **27** | İstanbul Sağlık Bilimleri Üniversitesi Mehmet Akif Ersoy Göğüs Kalp ve Damar Cerrahisi Eğitim ve Araştırma Hastanesi | Uzm. Dr. Ali Rıza Demir | PI |
|  |  | Dr. Yusuf Demir (left study at 11.04.2018) | SubI |
|  |  | Uzm. Dr. Emre Yılmaz | SubI |
| **28** | Erciyes Üniversitesi Tıp Fakültesi Hastanesi | Prof. Dr. Mehmet Tuğrul İnanç | PI |
|  |  | Dr. Bilge Bingöl | SubI |
| **29** | Selçuk Üniversitesi Selçuklu Tıp Fakültesi Hastanesi | Prof. Dr. Bülent Behlül Altunkeser | PI |
|  |  | Prof. Dr. Nazif Aygül | SubI |
|  |  | Dr. Muhammed Ulvi Yalçın | SubI |
| **30** | S.B. Yıldırım Beyazıt Üniversitesi Atatürk Eğitim ve Araştırma Hastanesi | Prof. Dr. Telat Keleş | PI |
|  |  | Doç. Dr. H. Ahmet Kasapkara | SubI |
|  |  | Dr. Serkan Bulguroğlu | SubI |
|  |  | Dr. Bilge Duran Karaduman | SubI |
| **31** | Koşuyolu Eğitim ve Araştırma Hastanesi | Doç. Dr. Taylan Akgün | PI |
|  |  | Dr. Ahmet Güner | SubI |
|  |  | Dr. Abdulrahman Naser (left study at 03.04.2019) | SubI |
| **32** | Tepecik Eğitim ve Araştırma Hastanesi | Doç. Dr. Öner Özdoğan | PI |
|  |  | Uzm. Dr. Murat Küçükukur | SubI |
| **33** | Recep Tayyip Erdoğan Üniversitesi Hastanesi | Doç. Dr. Ömer Şatıroğlu | PI |
|  |  | Yrd. Doç. Dr. Hakan Duman | SubI |
| **34** | Afyonkarahisar Sandıklı Devlet Hastanesi | Dr. İbrahim Etem Dural | PI |
